# TiO_2_ Thin Films Obtained via Two-Phase Dip-Coating: Impact on Surface Roughness and Application to Heterostructures

**DOI:** 10.1021/acsomega.5c09163

**Published:** 2026-02-10

**Authors:** Luiz Felipe Kaezmarek Pedrini, Natália Carli de Oliveira, Luis Vicente de Andrade Scalvi

**Affiliations:** † School of Sciences, Lab of Electro-Optical Characterization of Materials, Department Physics and POSMAT, 28108 São Paulo State University (Unesp), Bauru, São Paulo 17033-360, Brazil; ‡ School of Sciences, Department Chemistry, São Paulo State University (Unesp), Bauru, São Paulo 17033-360, Brazil

## Abstract

An innovative biphasic dip-coating process is introduced for the deposition of TiO_2_ thin films using a heterogeneous fluid system where an immiscible floating phase modifies the deposition dynamics. By controlling the height of the buoyant phase (Δ*H*), the method may reduce the agglomerate size during the gelation process, tending to shift the draining regime toward a capillary-dominated flow, a behavior typically observed only at lower withdrawal speeds. Numerical simulations based on Navier–Stokes equations suggest that increasing Δ*H* narrows and shifts the interface downward, which is consistent with the alteration in the stagnation point and deposition profile, supporting the role of pressure-driven flow and surface tension in deposition parameters. This controlled deposition mechanism may reduce the adhered precursor volume, leading to films with locally thinner deposited regions and decreased surface roughness. The proposed method suggests a direct correlation between floating phase height and film morphology, where an increase in Δ*H* is associated with smoother and more uniform thin films. This approach was applied to the development of a TiO_2_/SnO_2_ heterostructure, revealing via electrical characterization that heterostructures assembled with a thicker floating phase (Δ*H* = 0.6 cm) may exhibit higher homogeneity and reduced surface roughness, consistent with the behavior expected for a type-II heterojunction. The proposed biphasic dip-coating method presents a novel layering mechanism that may enhance film quality and provides a new parameter for adjusting thin-film properties, offering a promising alternative for advanced material processing in optoelectronics, sensors, and coatings.

## Graphical abstract

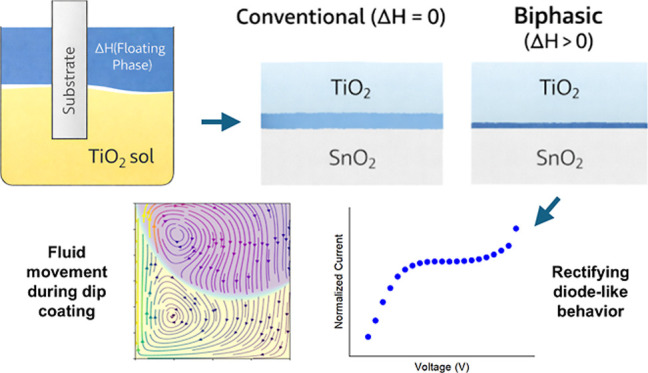

## 1 Introduction

Titanium dioxide (TiO_2_) is the most abundant naturally occurring form of titanium, which is predominantly found in its oxidized states.^1^ The widespread availability of this oxide, combined with its semiconductor properties, makes it a highly versatile material, with applications in gas sensors, solar cells, prosthetics, photocatalysis, among others.^2–7^

The properties of titanium dioxide strongly depend on its crystalline structure. Although many structures are reported,^8^ two crystalline forms are predominant in the literature: anatase (with an indirect band gap of about 3.4 eV) and rutile (with a direct band gap of 3.2 eV).^9–13^ To develop many kinds of devices, some parameters must be controlled, which affect the oxide structure. These include annealing temperature and duration, pH, pressure, and other factors.^14,15^ In this article, TiO_2_ thin films and TiO_2_/SnO_2_ heterostructure devices were prepared via sol–gel dip-coating. TiO_2_ samples are prepared using a two-phase stratified system of precursor solution (bulk phase) and a low-viscosity floating phase,^16–18^ while SnO_2_ depositions are prepared following the already established literature.^19^

The enhancement of TiO_2_-based heterostructures through metal oxide coupling has been widely explored in previous studies, particularly for their role in improving charge-transfer mechanisms and increased electrical conductivity due to surface defects.^19–21^ Although the functionality of such heterostructures is well-established, this study focuses on investigating how the deposition method influences the formation and properties of TiO_2_-based films, providing insights into the role of processing parameters in optimizing the heterostructure performance.

While this work focuses on an experimental approach, introducing a rather novel sol–gel dip-coating method for TiO_2_ thin films,^16^ the literature has developed further its theoretical models with Kumanan et al.^17^ modeling biphasic dip-coating flows on permeable substrates, deriving scaling laws and analyzing the effects of substrate permeability and slip conditions. Moreover, Champougny et al.^18^ have extended the classical Landau–Levich theory to two immiscible liquids, concluding that film thickness is primarily determined by interfacial shear stress and that the two liquid layers largely interact independently, contributing to the fundamental fluid mechanics of dip-coating.

This experimental approach modifies the draining regime, promoting a capillary-driven film formation process and enabling agglomerate size modulation, providing valuable insights into the interplay among fluid dynamics, phase separation, and thin-film uniformity.

Beyond its fundamental contributions, the biphasic dip-coating method holds practical implications for heterostructure fabrication, particularly in oxide-based electronic devices. By enhancing interface homogeneity, reducing roughness, and potentially influencing phase transition evolution during annealing, this method improves electrical conductivity and charge-transport properties in TiO_2_/SnO_2_ heterojunctions, making it a promising approach for optoelectronics, sensors, and flexible electronics. Additionally, its adaptability within the sol–gel deposition framework contributes to the controlled processing of diverse materials, broadening its potential across advanced coatings and thin-film applications.

## 2 Materials and Methods

The proposed dip-coating method incorporates a floating phase suspended over the precursor solution, introducing an additional hydrodynamic variable, the height of the floating phase, as a controllable parameter in the deposition process.

### 2.1 TiO_2_ Precursor Solution Synthesis Route

The precursor solution was developed through a derivation of methods by Hanaor et al.^22^ and Trino et al.^23^ To prepare a volume of 50 mL of the titanium dioxide precursor solution, the following reagents are used: 185.0 mL of deionized water obtained with a Millipore Milli-Q system, 56.7 mL of isopropanol (CH_3_CHOHCH_3_) (Merck), 2.6 mL of nitric acid (HNO_3_) (Synth), and 15 mL of titanium isopropoxide (TTIP) (Sigma-Aldrich 99,999%).

Deionized water and isopropyl alcohol are mixed under continuous magnetic stirring. Nitric acid is then slowly introduced to the solution to control the pH and facilitate hydrolysis. Once fully incorporated, TTIP is added dropwise, maintaining constant stirring for 30 min to ensure uniform precursor dispersion. The beaker is then covered with a single perforated sheet of aluminum foil to slow down solvent evaporation.

Unlike cited methods,^22,23^ this process introduces a floating phase by heating the solution to 120 °C until the final solution reaches 15 mL (instead of 50 mL), which can alter solvent evaporation dynamics. To ensure phase separation equilibrium, 5 mL of deionized water is added to the concentrated precursor solution, followed by sealing and resting until distinct phase separation is observed. The equilibrium time and the criteria for verification are discussed in the Results and Discussion section.

The floating phase height (Δ*H*) was controlled by pipetting excess water from the top layer or reintroducing the removed volume when needed. Adjustments are made before deposition to ensure consistent coating conditions. When not in use, the pipet liquid is stored in a sealed plastic container to prevent evaporation and maintain the compositional stability.

Δ*H* values were obtained using a caliper, by dipping its depth gauge and measuring the wetted regions on the millimeter scale. Both visual and numerical differences are clearly distinguishable and reproducible across samples.

A simplified diagram of the regular and the proposed stratified dip-coating is shown in Figure 1a,b, respectively, along with a picture of a sample solution in Figure 1c and a schematic of a finalized sample in Figure 1d.

**Figure 1:**
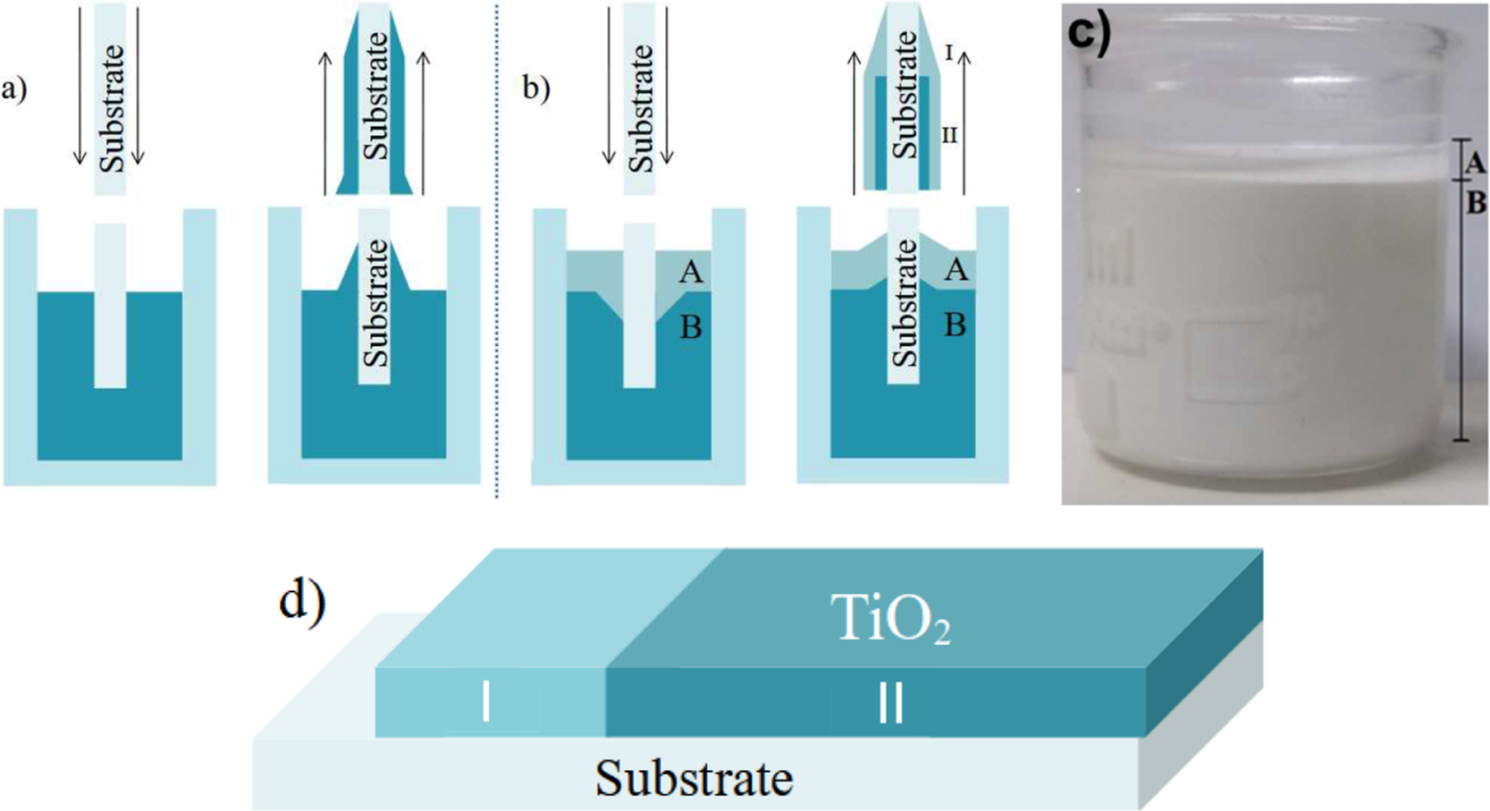
Comparison between (a) conventional dip-coating, (b) developed method, (c) image of the developed system, and (d) diagram of the resulting film.

The present study focuses mainly on denominated region II, where the substrate is directly exposed to both the precursor and floating phases.

### 2.2 Sample Preparation

Soda-lime glass substrates (10 × 25 × 1.0 mm, from Knittel Glass) are first immersed for 24 h in a 9:1 solution of deionized water and neutral detergent (Extran) to remove contaminants. After this soaking period, they are rinsed in deionized water for 5 min and subsequently bathed in isopropyl alcohol for 5 min more and are rapidly dried using a thermal blower.

For film deposition, the cleaned substrates are secured in a substrate holder connected to a Syringe Pump system (Model MQBSG 1/302) with a controller (Model MQCTL 2000 MP, both from Microchemistry). The precursor solution is placed in a beaker beneath the substrate holder, and deposition is carried out via dip-coating at a fixed withdrawal and immersion rate of 10 cm/min under ambient conditions (25 °C and 1 atm).

The coated substrate is set to drain and dry for 10 min and then placed on a ceramic base and introduced into an EDGCON 3P oven at 150 °C for 10 more minutes. Following this treatment, the film is left to cool to room temperature before being reinstalled in the substrate holder for additional depositions.

All intermediate thermal annealing processes were performed in the same EDGCON 3P forced-convection oven using a ceramic holder. Samples were inserted at a set temperature and removed to ambient air after each step.

After the desired number of deposited layers is achieved, a final annealing process is conducted. In this step, the sample is heated gradually from room temperature at a constant rate of 3 °C/min until reaching 500 °C, where it is maintained for 2 h to ensure full crystallization and phase stabilization.

The entire process was performed in air at room temperature and atmospheric pressure without the use of controlled or inert environments.

Table 1 provides an overview of the prepared thin films and the characteristics of the two-phase solution used in the deposition process, with Δ*H* referring to the height of the floating phase.

**Table 1:** TiO_2_ Film Samples Prepared According to the Two-Phase Method

Δ ***H*** (cm)	# of layers	sample name
0.0 (removed)	1	**1L_0**
	2	**2L_0**
0.3	1	**1L_3**
	2	**2L_3**
0.6	1	**1L_6**
	2	**2L_6**

Heterostructures were prepared by depositing TiO_2_ onto SnO_2_ films, where SnO_2_ was deposited using conventional sol–gel dip-coating, using the sol prepared from SnCl_4_-5H_2_O as described elsewhere,^19^ and only TiO_2_ was deposited using the proposed biphasic method.

The SnO_2_ films were prepared through 10 successive layer depositions, each followed by an intermediary heating step at 400 °C for 10 min (instead of the 150 °C used for TiO_2_). After each 400 °C/10 min bake, samples were cooled to room temperature on the benchtop for 20 min before the subsequent dip-coat. After completing all 10 layer depositions, the films underwent a final thermal annealing treatment at 550 °C for 2 h, with a controlled heating rate of 3 °C/min. The same deposition setup and withdrawal speed used for TiO_2_ films were applied to the SnO_2_ films to maintain consistency in the process.

### 2.3 Characterization Methods

Scanning electron microscopy (SEM) measurements were performed using a Carl Zeiss scanning electron microscope, model LS15. The analysis was conducted with an accelerating voltage (EHT) of 10.00 kV and a working distance of 9.5 mm. The SE1 detector was used to capture secondary electrons emitted close to the surface. Images were acquired at a magnification of 4000×, enabling detailed visualization of surface microstructures. Complementary morphological characterization was carried out using confocal microscopy on a Leica DCM 3D system equipped with high-power white (emission centered at 530 nm) and blue (460 nm) LED light sources, allowing for three-dimensional surface profiling and optical contrast enhancement.

The film’s optical properties were observed through optical transmittance and reflectance from the ultraviolet to the near-infrared region (250 – 1800 nm) in a Lambda 1050 UV/vis/NIR PerkinElmer spectrophotometer with a scanning rate of 141 nm/min.

X-ray diffraction (XRD) measurements were carried out using a Rigaku Miniflex 600 diffractometer, operated at 40 kV and 15 mA, employing Cu Kα radiation (λ = 1.54056 Å) as the X-ray source. A nickel (Ni) filter was used to suppress unwanted Kβ radiation. Diffraction patterns were recorded in the 2θ range of 10°–80°, with a step size of 0.04°, ensuring high-angular resolution for phase identification and structural analysis.

Electrical characterization of the TiO_2_ films and the SnO_2_/TiO_2_ device was accomplished through current as a function of the applied voltage, at distinct temperatures, using a Janis Research He gas closed-circuit cryostat coupled to a CTI-Cryogenics compressor, and of a Lake-Shore temperature controller (sensor in thermal contact with the substrate holder; stability, ±0.1 K). The biasing and collection of electrical current were performed with the aid of Keithley electrometer model 6517A. Measurement sets were recorded after 5 min stabilization at the target temperature; typical ramp rate was 5 K min^–1^.

## 3 Results and Discussion

### 3.1 Morphology and Structure

Figure 2 presents an XRD pattern of a TiO_2_ thin film, prepared by the same solution, from the bulk phase and without a floating phase (Δ*H*). The diffraction peaks in the black curve correspond to the crystalline phases present in the sample. The reference peaks for anatase (red dashed lines) and rutile (blue dashed lines) phases are included for comparison.

**Figure 2:**
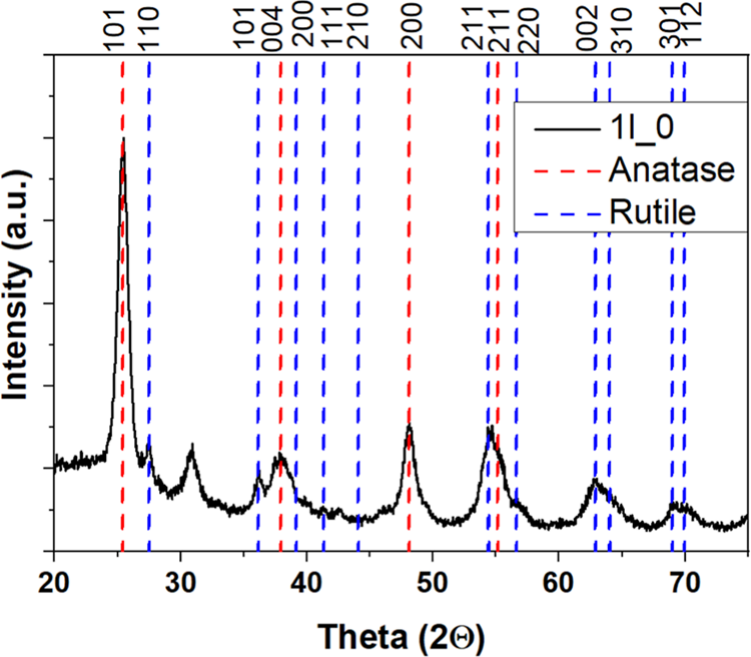
XRD pattern of a TiO_2_ thin film (1l_0) showing anatase and rutile phases.

From the observed peaks, it is inferred that the film exhibits a dominant anatase phase, indicated by the strong diffraction at 2θ ≈ 25° (anatase (101)), along with other characteristic peaks of this phase.^9^ However, the presence of some peaks aligning with the rutile phase, particularly at higher angles, suggests the possible coexistence of anatase and rutile phases, based on qualitative peak matching with reference patterns.^24^

Although XRD provides qualitative phase identification, it does not allow for the direct assessment of agglomerate size or transformation kinetics. Nevertheless, previous studies^24^ have shown that particle size, surface energy, and colloidal stability strongly influence the anatase–rutile transition in sol–gel-derived TiO_2_ systems. In the present work, these mechanisms are invoked as a plausible interpretative framework rather than being directly demonstrated by the diffraction data. When the precursor solution leads to the formation of smaller agglomerates, the increased surface area-to-volume ratio and reduced diffusion pathways favor the nucleation and stabilization of the anatase phase, resulting in anatase-dominant films.^24^ However, at even smaller agglomerate sizes, the anatase-to-rutile transition can occur earlier at lower annealing temperatures due to the higher surface energy and increased nucleation sites for rutile crystallization. This transition at lower temperatures, attributed to the higher surface energy of fine particles, is also related to the increased availability of nucleation sites for rutile crystallization. The destabilization of colloids, often induced by ionic species in the precursor solution or a decrease in pH, promotes the formation of smaller, less cohesive agglomerates consistent with previous studies,.^24–26^ These conditions enhance the thermodynamic instability in the anatase phase, lowering the temperature threshold observed for the anatase–rutile transition.

The surface morphology and thickness of the TiO_2_ films were investigated using SEM and confocal microscopy, with particular attention to the distinct deposition settings defined as regions I and II, according to Figure 1. Region I corresponds to the portion of the substrate exposed exclusively to the floating phase during withdrawal, while region II is exposed to both the precursor solution and the floating phase. This distinction is essential for interpreting morphological features, thickness measurements, and their correlations with electrical transport properties.

Figure 3 shows SEM images of region 1 of films prepared with two deposited layers that vary in the floating phase thickness (Δ*H*), as it was called by Champougny et al.^18^ and Kumanan et al.^17^ Refer to Table 1 for sample names and deposition parameters.

**Figure 3:**
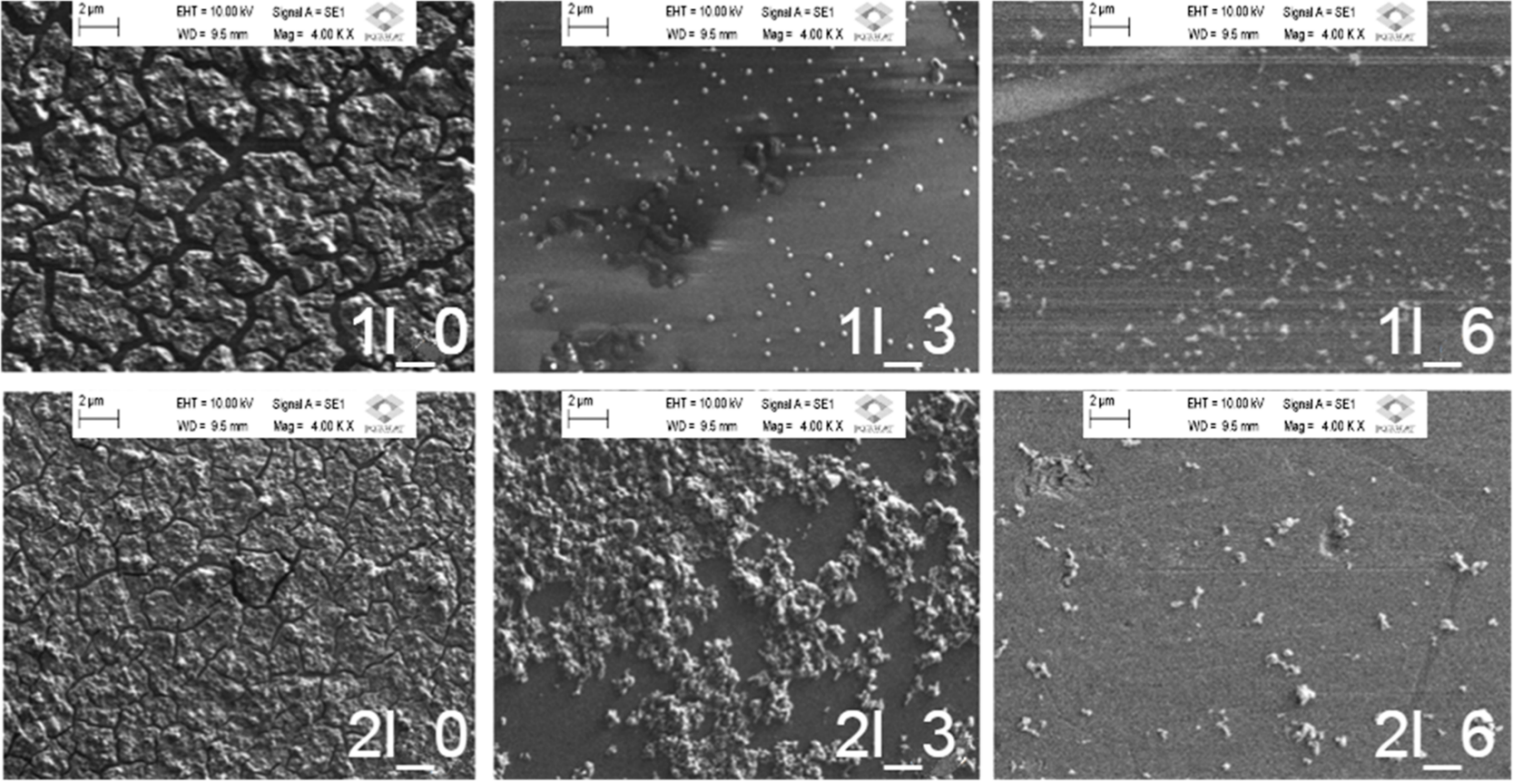
SEM images from region I of films deposited with distinct heights of the floating phase layer. Magnification: 4000x.

Although this study primarily focuses on region II for thickness and transport analysis, region I provides direct insights into the influence of the floating phase on the local deposition behavior, material retention, and morphological continuity.

The SEM images shown in Figure 3 indicate that in region I, increasing Δ*H* leads to a progressively sparser deposition, with the material forming isolated island-like features rather than a laterally continuous film. The deposited material shows sparse “islands”, not forming a consistent and homogeneous thin-film structure. These SEM images of region I are consistent with the explosion only to the floating phase and therefore exhibit reduced material retention and island-like morphologies at higher Δ*H*. In contrast, thickness measurements and electrical analyses were performed exclusively in region II, where the substrate is exposed to both phases and material continuity is preserved.

Figure 4 shows surface confocal images of region II of samples listed in Table 1.

**Figure 4:**
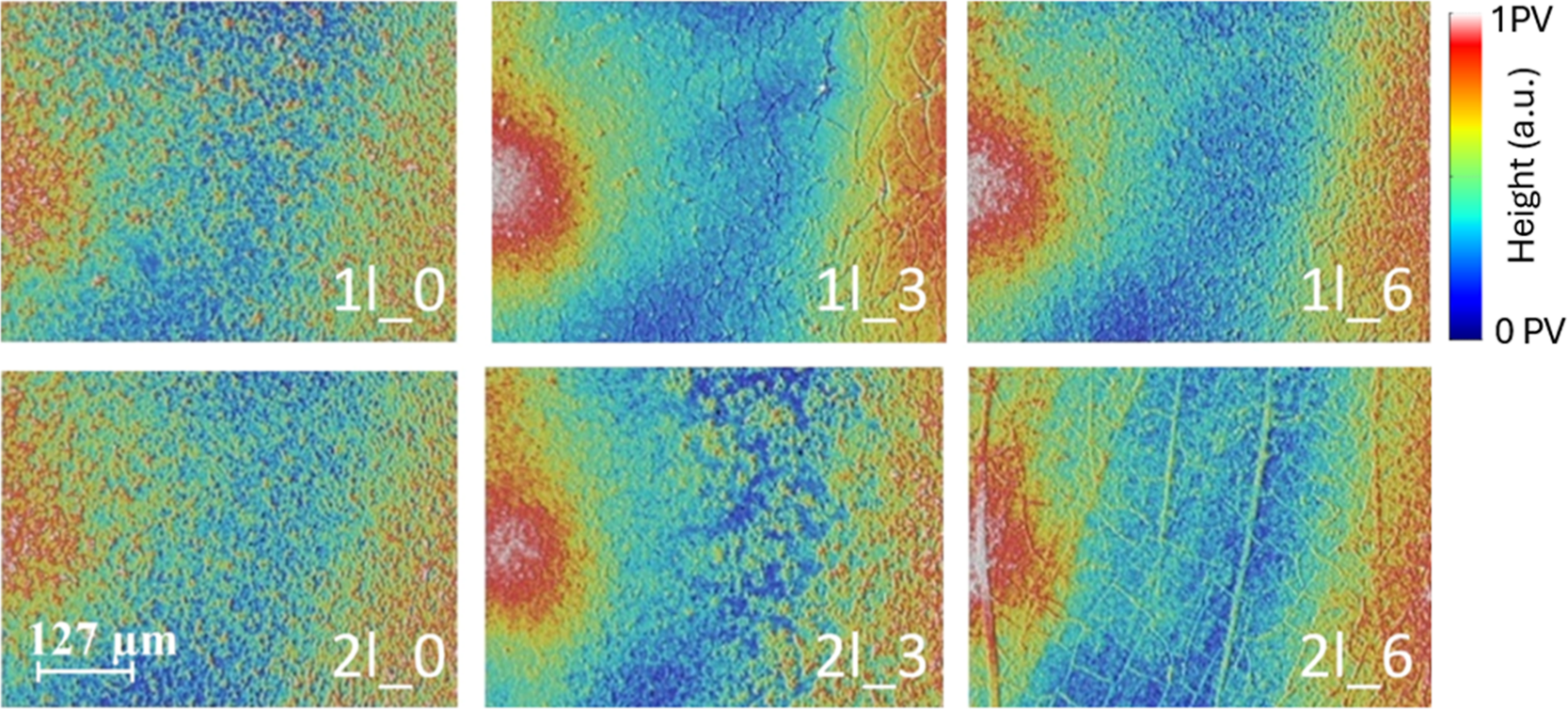
Confocal images of samples in region II surface with distinct heights of the top phase and number of layers. Samples characteristics are listed in Table 1, and images shown are 636 × 477 μm.

Film thickness is determined by mechanically scraping the surface of the film with a steel-point prod to remove the film and expose the underlying substrate. The thickness is then measured by profiling across the grooves left behind, allowing for a direct and reliable measurement of the film thickness. Data are shown in Figure 5.

**Figure 5:**
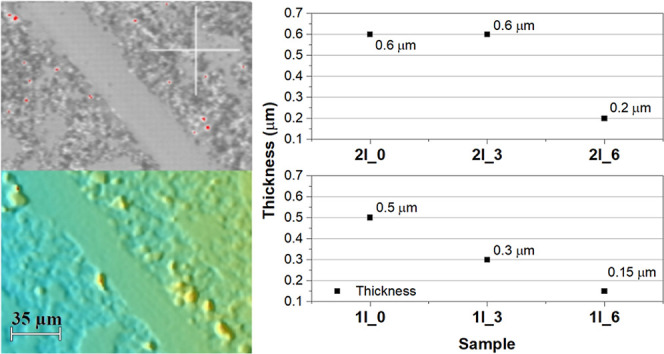
(Left) Example of a groove observed under a confocal microscope with 20× magnification (sample 1l_6). Images shown are 177 × 144 μm. (Right) Estimated thickness of all samples.

Due to surface roughness and morphological heterogeneity, particularly for discontinuous films, thickness measurements carry an estimated uncertainty on the order of ±10–20%, which does not affect the observed qualitative trend of decreasing retained material with increasing Δ*H*; the observed decrease in film thickness remains consistent across both the series of samples. Regardless of whether the deposition is repeated or singular, the overall trend indicates a reduction in thickness, suggesting that the material retention on the substrate is affected by deposition conditions. However, the degree of this reduction varies depending on Δ*H* (floating phase height), indicating that interface dynamics, drainage efficiency, and the precursor solution behavior play a role in controlling film formation and material adhesion. Such a control possibility suggests that higher Δ*H* enhances solvent drainage or reduces the volume of entrained material, leading to locally thinner deposited regions and reduced areal coverage with thinner local features, but the extent of this effect differs across different deposition conditions.

Figure 6 presents the data acquired by confocal microscopy from the surfaces of the thin-film samples. The data suggest a nonlinear relationship between Δ*H* (floating phase thickness) and surface roughness, with an initial increase in roughness from Δ*H* = 0.0 to Δ*H* = 0.3 cm, followed by a decrease from 0.3 cm to Δ*H* = 0.6 cm, a behavior that aligns with the plateau effect described by Champougny et al.,^18^ where the contribution of the floating liquid phase to deposition saturates beyond a certain threshold, and entrained liquid from the floating phase does not change.

**Figure 6:**
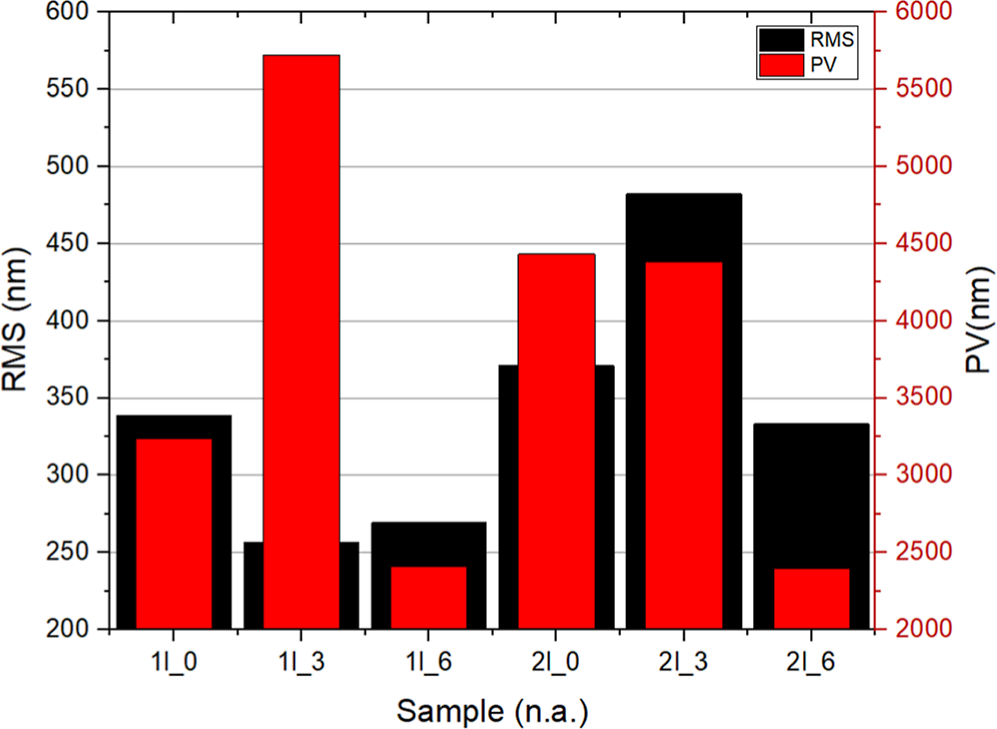
Roughness (RMS) and height variation (PV) obtained from confocal microscopy data.

During substrate removal, the floating phase exerts pressure on the liquid/liquid interface, reducing the volume of the deposited material by decreasing the amount of bulk liquid entrained. This behavior has been observed in previous studies^16^ and aligns with that of Champougny et al.,^18^ which describe how increasing Δ*H* pushes the lower liquid/liquid interface downward. At a low capillary number (Ca), the entrained floating phase increases, as it is drawn upward along the substrate. Consequently, a higher Δ*H* shifts the stagnation point closer to the bulk liquid (denser phase) and the substrate, altering the deposition process.

This effect explains the nonlinear roughness variation observed in the films. The increase in roughness at Δ*H* = 0.3 cm likely results from the fluctuations in liquid entrainment, while the decrease at Δ*H* = 0.6 cm corresponds to a reduction in the deposited material due to greater pressure from the floating phase, which reinforces the idea that with increasing Δ*H*, the stagnation point moves upward, bringing the interface closer to the substrate, affecting deposition uniformity.

Surface roughness tends to increase with more layer depositions, regardless of Δ*H*, as pre-existing surface imperfections serve as nucleation sites for further growth. This effect becomes more pronounced in the proposed method, suggesting a stronger influence of deposition dynamics on the film morphology.

Fixation of the material occurs after the substrate emerges from the precursor solution. Considering a smaller volume of the bulk solution entraining to the substrate, small-particle agglomerates are more likely to attach to the surface of previously deposited layers rather than remain suspended in the solvent, which takes place due to the existing film that provides more favorable fixation sites compared to dispersed particles in the liquid phase.^27^

Given the limited material deposited per deposited layer and the reduced solvent volume present in the drying film, particles suspended in the solution have two primary growth pathways before heating: (1) agglomeration within the liquid phase or (2) fixation onto the substrate of previously deposited layers in a multilayer sample. These processes are not mutually exclusive, but one of them may dominate depending on the deposition conditions, particle interactions, and solvent dynamics.^28–30^

With this reduction in entrained volumes, something akin to a transition of draining regime may be identified that suggests a shift in the dominant deposition mechanism at higher Δ*H*, where capillary draining channels on the sample surface provide attachment and nucleation points. The results indicate that the moderate floating phase heights (Δ*H* = 0.3 cm) promote surface irregularities due to increased liquid entrainment, while a further increase to Δ*H* = 0.6 cm results in a more stabilized film structure, supporting the plateau behavior predicted in the biphasic dip-coating models, which can be responsible for the increases and decreases in roughness observed.

As the volume of adhered solution during the dip-coating process is reduced, the rate at which particles actually adhere to the already deposited material increases, which leads to sequential depositions to elevations and rougher surfaces.^16^ Rougher surfaces lead to an increased flow of solution, changing further the main draining regimen to the capillarity fed.^28^ This results in further accumulation of material in the regions of the sample that already present agglomeration of the deposited material. Figure 7 is a simplified flow diagram of how both draining regimes behave and how this difference in behavior can lead to different topologies.

**Figure 7:**
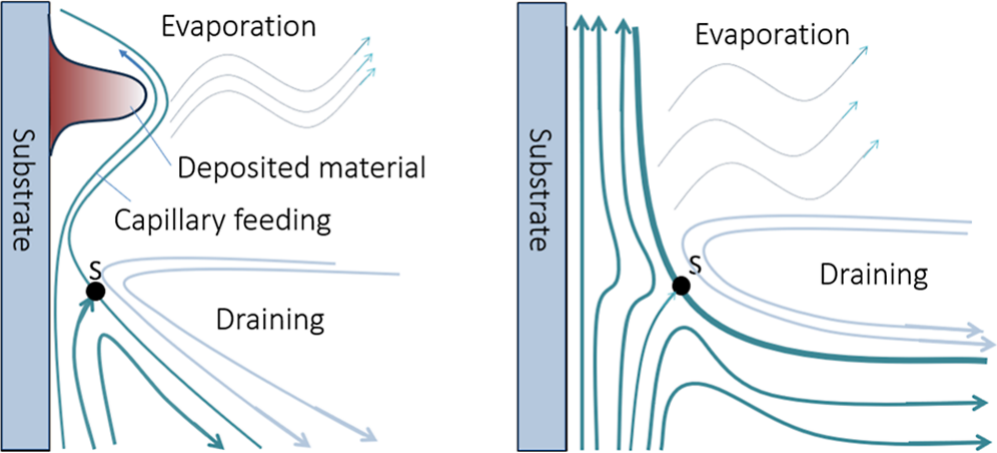
Simple diagram showing distinct regimes of draining in the film growth, based on Brinker.^31^

Figure 8 illustrates how an increase in Δ*H* may lead to a decrease in agglomerate size. The floating phase leading to a lower volume of entrained precursor solution, compressing the liquid layer on the substrate, may reduce the overall amount of deposited material. As the height of the floating phase increases, this effect becomes more pronounced, leading to further thinning of the deposited layer. This behavior is shown in the modeling section and is displayed in Figure 10.

**Figure 8:**
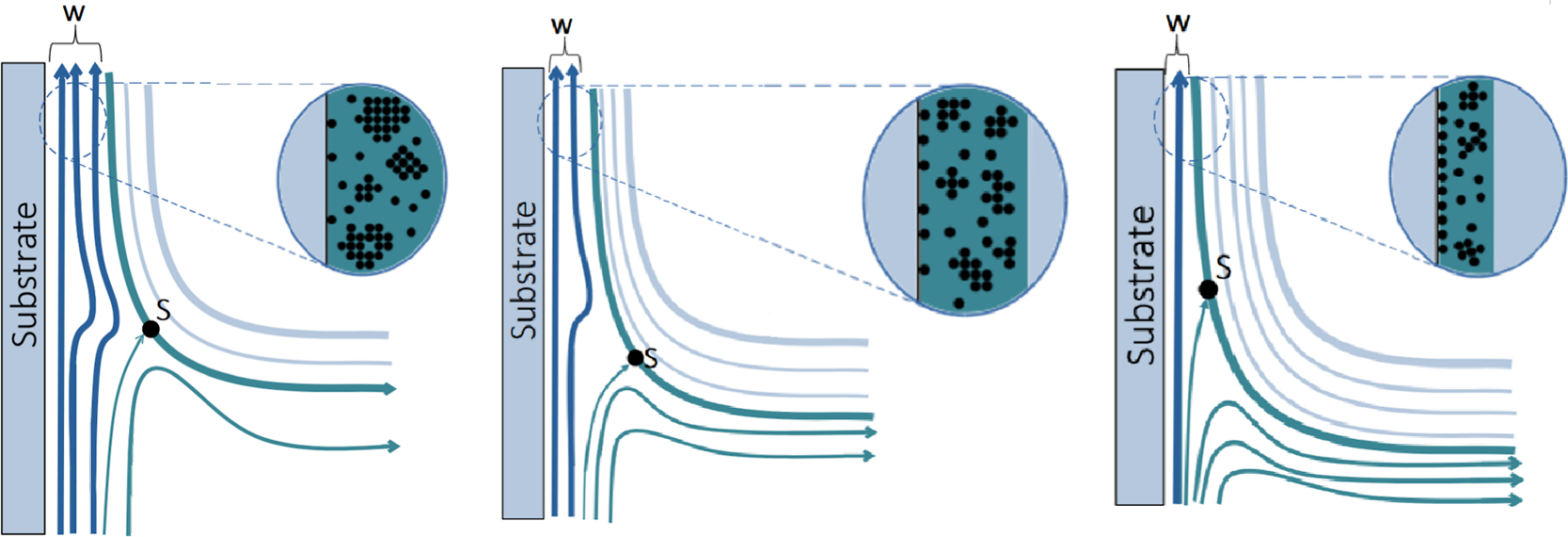
Diagram showing the increase of volume of the superior phase, leading to reduction of the mean agglomerate size during gelification.

As illustrated in Figure 8c, increasing Δ*H* may reduce the volume of the entrained precursor solution, limiting agglomerate growth during evaporation and gelation and promoting the fixation of smaller agglomerates onto the substrate. This possible behavior is consistent with the roughness trends in Figure 6, where intermediate Δ*H* (0.3 cm) yields higher roughness, while higher Δ*H* (0.6 cm) leads to smoother surfaces, reflecting the thinning of the entrained bulk phase (*w*). While such conditions may favor improved local interfacial coupling and have previously been associated with enhanced photocatalytic activity in TiO_2_ films, Δ*H* should not be regarded as an independent control parameter. Rather, it acts as a hydrodynamic variable that modulates liquid entrainment, drainage efficiency, and the amount of material retained during withdrawal, with concomitant effects on local thickness, continuity, and surface coverage. It was previously observed that the method proposed in this work increases the photocatalytic capacity of TiO_2_ films.^16^

### 3.2 Optical Characterization

Figure 9 displays transmittance and reflectance data for the samples listed in Table 1. During crystallization, the formation of a high density of grain boundaries becomes a key factor in reducing transparency due to light scattering.

**Figure 9:**
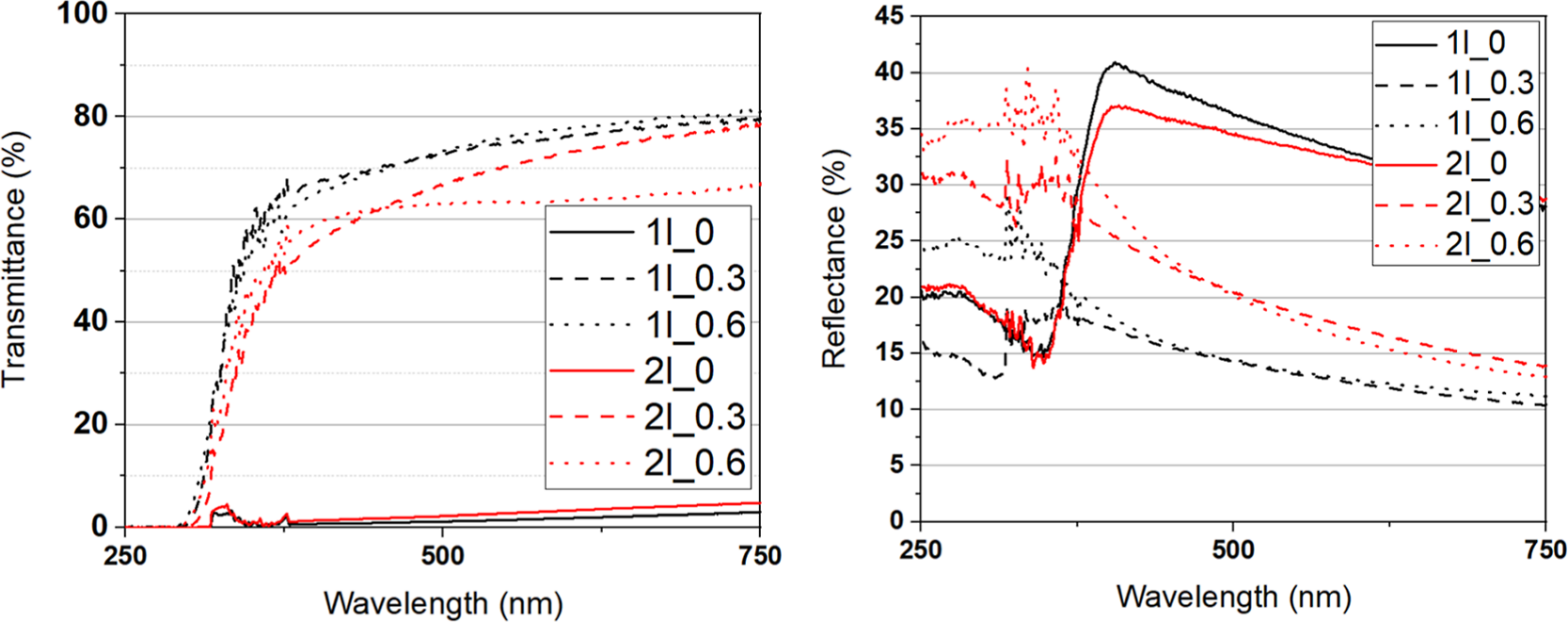
Optical transmittance (left) and reflectance (right) for films listed in Table 1.

As shown in Figure 9 (left), samples prepared with Δ*H* = 0 exhibit lower transparency compared to those with Δ*H* > 0. Additionally, samples with multiple deposited layers display a slight reduction in transparency compared to those with a single-layer deposition, indicating the influence of film thickness on optical properties.

The reduced transparency in Δ*H* = 0 samples may be correlated with their higher reflectance, as observed in Figure 9 (right), and can be attributed to the larger volume of deposited material, leading to more internal reflecting surfaces and an increased number of crystal interfaces, which further scatter light and contribute to optical losses.

### 3.3 Flow Morphology

Based on Carvalho et al.,^32^ a model to simulate and study the proposed method was developed here and established on how the fluid dynamics govern film entrainment. The domain is a rectangle mesh of cells of dimensions 256 × 256, with a vertical substrate at the left boundary withdrawn upward at a speed *U*_w_.

To emulate the proposed method, a one-fluid formulation is attempted: a smooth indicator ϕ ∈ [0,1] denotes the local volume fraction of liquid A (liquid B has the fraction 1 – ϕ).

The mixture density (ρ) and viscosity (μ) of each cell are then calculated as

1 $$\rho \left(\phi \right)=\phi \rho_{A}+\left(1-\phi \right)\rho_{B}$$

2 $$\mu \left(\phi \right)=\phi \mu_{A}+\left(1-\phi \right)\mu_{B}$$

To isolate the behavior of the proposed dip-coating method, a third phase (air) is omitted, and evaporation, molecular-scale wetting, and detailed interfacial chemistry are not modeled. In particular, the present model deliberately excludes evaporation, gelation kinetics, and non-Newtonian rheology, processes that are known to be important in many sol–gel systems where drying and structural evolution control the final film properties.^33,34^ Similarly, effects such as dynamic contact angles, thixotropic rheology, and nanoscale or electrostatic interactions^35–37^ are considered outside the scope of this approach. However, despite these limitations, the simulation captures a qualitative fluid behavior hypothesized under varying Δ*H* conditions.

The flow is mapped on a Marker and Cell grid using a finite-difference method.^32^ At each step: (i) the velocity and the composition indicator ϕ are calculated, (ii) cell forces (capillarity and gravity) are added, (iii) viscous diffusion, mainly at the interface, is applied, and (iv) a pressure equation, binding compressibility, before correcting velocities is solved. Composition (ϕ) denotes the volume fraction of liquid B, where ϕ = 0 means pure A and ϕ = 1 means pure B; intermediate values occur only across the interface (a few cells thick). The interface is slightly regularized by a small diffusivity, *D*_ϕ_, in eq 3, which adds minimal smoothing without affecting the bulk phases:

3 $$\partial_{t}\phi +u\cdot \nabla \phi =D_{\phi }\nabla^{2}\phi$$

The capillary number (*Ca*) is given by^32^

4 $$Ca=\frac{\mu_{A}U_{w}}{\sigma_{AB}}$$

and the Reynolds (*Re*) number is^32^

5 $$Re=\frac{\rho_{A}U_{w}L}{\mu_{A}}$$

To establish consistent parallels with empirical data, within this framework, the formation and migration of the stagnation point and changes in streamline topology as Δ*H* is varied are tracked. Data used for the simulation and their experimental counterparts are given in Table 2.

**Table 2:** Values for the Floating Phase (A) and Bulk Phase (B)^a^

quantity	expression	experiment	model
withdrawal speed	U_w_	0.01	0.01
viscosity (floating)	μ_A_	∼0.01	0.01
viscosity (subphase)	μ_B_	∼0.03	0.03
density (floating)	ρ_A_	∼1000	1000
density (subphase)	ρ_B_	∼1000	1100
gravity	g	9.81	9.81
capillary number (A)	*Ca*_A_ = μ_A_ *U*_w_/σ_AB_	∼0.004	0.005
capillary number (B)	*Ca*_B_ = μ_B_ *U*_w_/σ_AB_	∼0.01	0.01
Reynolds (A)	*Re*_A_ = ρ_A_ *U*_w_ h/μ_A_	∼1.87	∼5
Reynolds (B)	*Re*_B_ = ρ_B_ *U*_w_ h/μ_B_	∼5	∼5
contact angle (A on wall)	θ_A_	∼20	∼20

a Experimental data are reported as nondimensional parameters (e.g., *Ca*, *Re*, θ). Model parameters are selected to be of the same order of magnitude as the experimental conditions.

Figure 10 shows the early evolution of the liquid–liquid interface under four conditions: (a) *U*_w_ = 0, (b) large Δ*H*, (c) intermediate Δ*H*, and (d) small Δ*H*. Left panels give the initial state at *t* = 0 (indicator ϕ: yellow = bulk-phase B, purple = floating phase A); right panels are snapshots at comparable times with streamlines colored by speed magnitude. Boundary conditions are a vertically moving substrate on the left (*v* = *U*_w_), closed bottom and right walls, and an open top. The baseline interfacial behavior was first established with *U*_w_ = 0; then, finite pool depths by setting the initial height of liquid A to Δ*H*/*L*_*y*_ = {1/4, 1/2, 3/4} are introduced. The analysis utilizes the values shown in Table 2 to better show what develops in the empirical configuration. The key dimensionless groups, primarily the capillary number and the Reynolds number, constrain parameters with regard to the real values.

**Figure 10:**
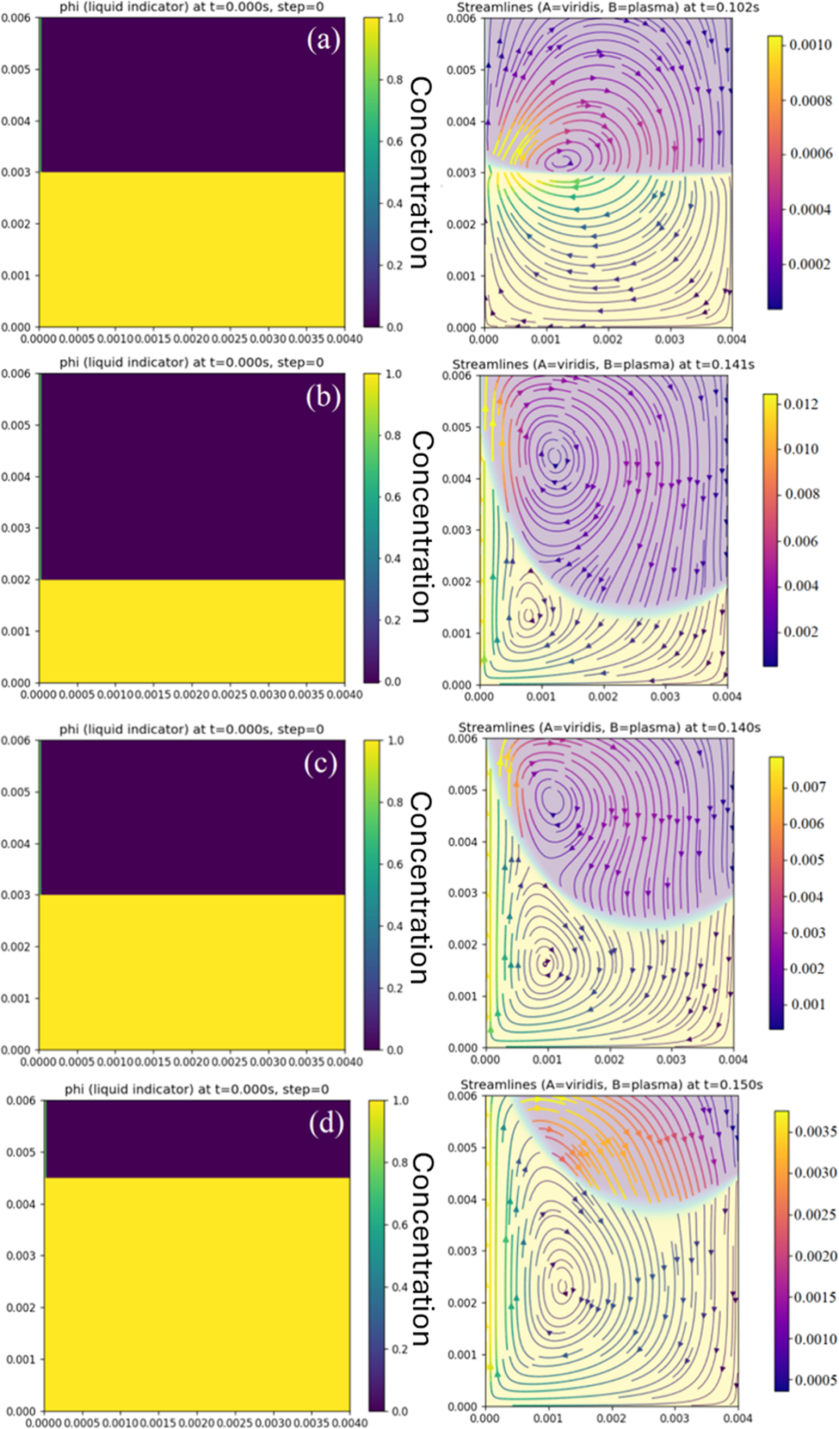
Evolution of a liquid–liquid meniscus under different Δ*H* and wall speed *U*_w_. Left panels: indicator field ϕ (liquid A fraction) and how the fluids are started in the simulation (*t* = 0 s). Right panels: streamlines colored by speed magnitude after the development of the stagnation point. (a) *U*_w_ = 0 (immobile substrate): static capillary meniscus forms; no entrainment. (b) Large Δ*H.* (c) Intermediate Δ*H.* (d) Small Δ*H*.

The elapsed time Δ*t* of 0.140 s was chosen, as the simulation has shown to stabilize the position of the stagnation point.

With *U*_w_ = 0, the system relaxes to a static capillary meniscus and negligible circulation. As Δ*H* decreases, the meniscus shoulder shortens, and a stagnation point emerges sooner and shifts toward the substrate, concentrating flow near the wall and reducing the volume entrained into the film.

Higher Δ*H* gives the interface more space to curve and flow into the adhered layer that shears off and back into the solution; therefore, the returning flow develops around a bigger area close to the stagnation point, which also creates a region of much faster flow near the substrate. Lower Δ*H* makes the interaction between the substrate and bulk solution a lot quicker, so the adhered flow starts earlier and develops a split between film-entrained and returned solution sooner. When the stagnation point approaches the left wall, a smaller flow is directed toward the substrate surface, and more fluid returns to the precursor solution along the interface.

### 3.4 Electrical Characterization

The electrical characteristics of the prepared films were investigated through indium contacts deposited by resistive evaporation onto their surface to make ohmic contacts for TiO_2_ films, allowing electric polarization of the sample. Electrical measurements probe charge transport through region II of TiO_2_ samples, where material continuity is preserved and improved local uniformity and reduced roughness are observed.

Figure 11 presents the plots of current as a function of applied voltage for the different films, as listed in Table 1.

**Figure 11:**
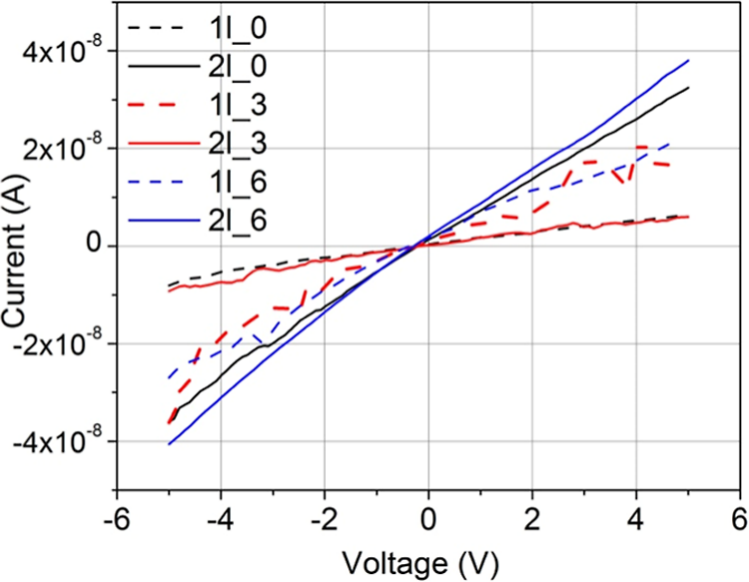
Current as a function of applied voltage for all the samples listed in Table 1.

An increase in conductivity is observed with a higher number of layers for samples deposited with Δ*H* = 0; however, this trend does not hold for films with Δ*H* > 0. Specifically, in films prepared with Δ*H* = 0.3 cm, conductivity decreases with additional deposited layers. The analysis of the surface morphology via confocal microscopy (Figure 6) suggests reducing the overall deposited material and promoting island-like regions of material with thin conduction channels between them. This morphology also correlates with a decrease in transmittance and an increase in reflectance, as the expanded surface area would enhance light scattering (Figure 9).

For films prepared with Δ*H* = 0.6 cm, conductivity increases again with additional deposited layers. This improvement is attributed to film densification, as previously discussed, as samples with Δ*H* = 0.6 cm exhibit a decrease in RMS roughness, whereas PV values remain similar for both single- and double-layer depositions. In samples prepared with Δ*H* = 0.3 cm, this reduction enhances surface roughness, as altered drainage conditions favor vertical columnar growth.

The observed roughness suggests that a further increase in Δ*H* reduces both the deposited material and solvent volume, leading to smaller agglomerate sizes during gelation. This size reduction affects multiple film properties, as smaller agglomerates are more likely to fix onto the substrate due to the lower energy barrier required for migration and attachment. The result is a denser, more homogeneous film, as seen in Figure 6, which shows a smoother surface, and in Figure 11, where improved uniformity correlates with enhanced electrical conductivity.

### 3.5 TiO_2_/SnO_2_ Heterostructure

The impact on material interfaces of the proposed deposition method has clear applicability in heterostructures. Then, the coupling of TiO_2_ layers, prepared by this method, with SnO_2_ layers was done to assess the impact of material interfaces on the heterostructure performance.

The TiO_2_/SnO_2_ heterostructure has been extensively studied for applications in sensing technologies and pollutant removal.^21,29,30^ Heterojunction formation, with application in electronics, is possible when both materials are produced in the rutile phase, where their lattice parameters differ by approximately 5%.^11^ The energy band diagram of the TiO_2_/SnO_2_ heterostructure is shown in Figure 12 (left).

**Figure 12:**
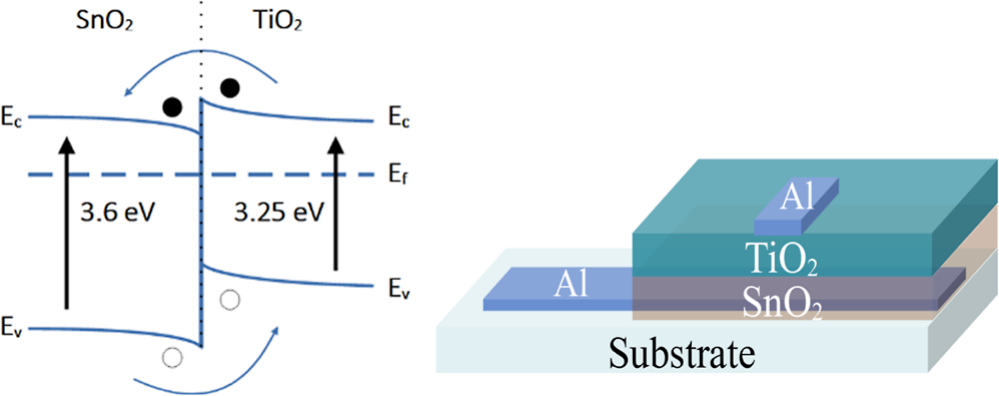
(Left) Energy band diagram of TiO_2_ and SnO_2_ showing the possible way of charge carrier movement at the interface, electrons (filled circles), and holes (empty circles). (Right) Heterostructure sample diagram.

For device assembly, SnO_2_ was first deposited as the bottom layer using the conventional dip-coating technique,^21^ followed by the deposition of TiO_2_ by the method presented here. Aluminum contacts were added via resistive evaporation and placed beneath the SnO_2_ layer (on the substrate) and above the TiO_2_ layer, establishing a conductive channel across both materials and their interfaces. A schematic representation of the device is provided in Figure 12 (right).

The behavior described in Figure 12 can be used to interpret data from heterostructures TiO_2_/SnO_2_ produced by the two-phase method. Figure 13 represents current as a function of applied voltage, measured in the range 100–300 K, for samples with a distinct height of the top phase of TiO_2_ solution (Δ*H*), as described in the Materials and Methods section (Table 1).

**Figure 13:**
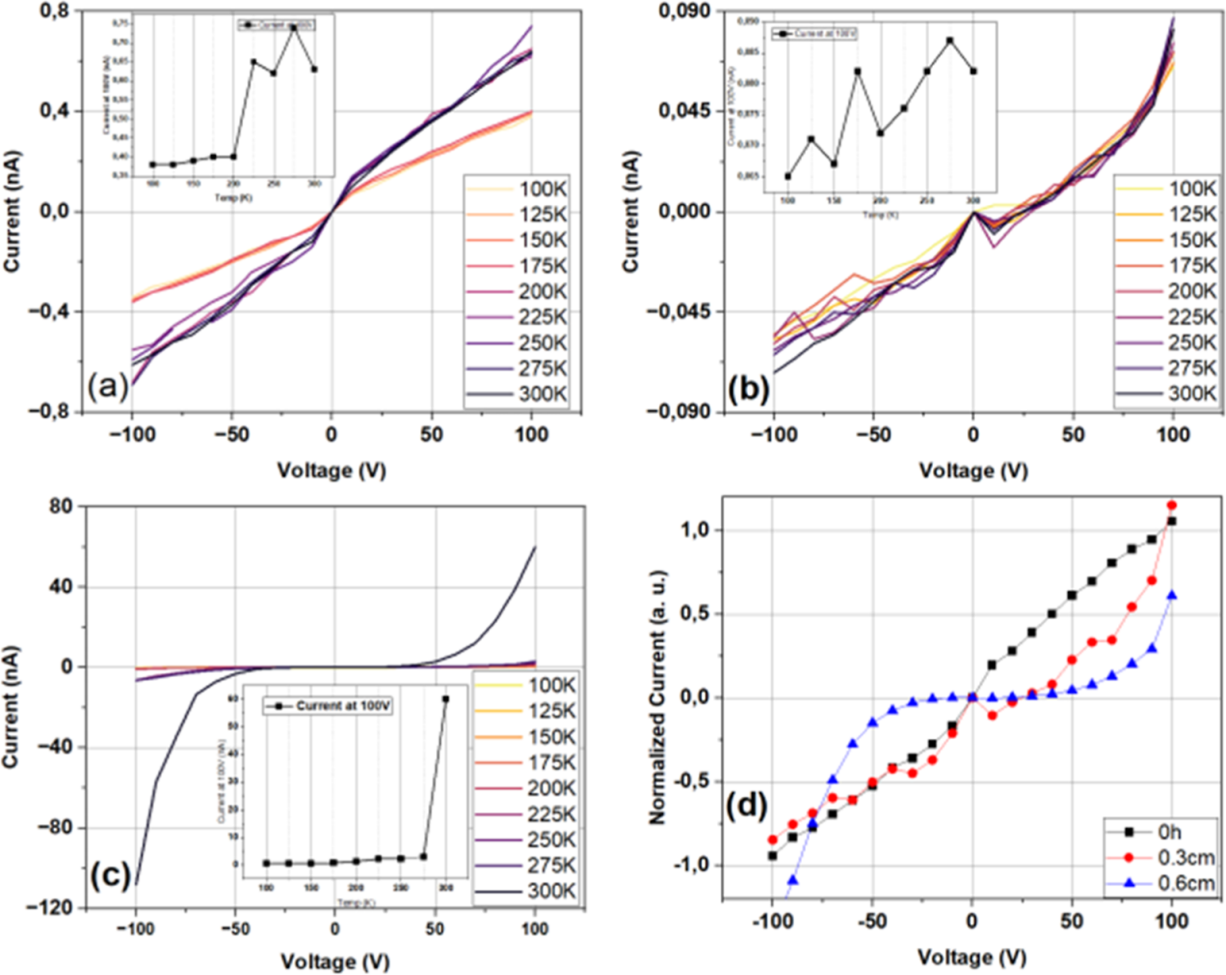
Current as a function of applied voltage for heterostructures TiO_2_/SnO_2_ for distinct heights of the top phase in TiO_2_ layer deposition, measured at distinct temperatures (from 100 to 300 K). (a) Δ*H* = 0; (b) 0.3; (c) 0.6 cm. Insets represent current observed at 100 V as a function of sample’s temperature. (d) Normalized current–voltage comparison among data measured at room temperature.

Heterostructures fabricated from solution with Δ*H* = 0.3 cm exhibited linear current–voltage (*I*–*V*) characteristics over a broad temperature range (100–300 K), as can be seen in Figure 13, consistent with the ohmic behavior. In contrast, devices prepared using the modified biphasic deposition method with Δ*H* = 0.6 cm displayed nonlinear *I*–*V* profiles that are stable across the same temperature range, indicative of rectifying behavior, which is consistent with type-II heterojunctions. This nonlinearity, particularly in isotype n–n oxide systems such as TiO_2_/SnO_2_, may arise from staggered (broken) band alignment at the interface, which spatially confines minority carriers (holes) while facilitating electron flow.^38^ This band misalignment promotes asymmetric transport and carrier separation, features commonly associated with the type-II heterostructure, and is fundamental to optoelectronic device functions such as photonic memory, synaptic response, and optical communication.^39^

In the case of the Δ*H* = 0.6 cm heterostructure, the current increases exponentially with temperature, as can be seen in the inset of Figure 13c, indicating a thermally activated transport process. This behavior is consistent with junction-mediated conduction, where charge transport is limited by an interfacial potential barrier that can be more efficiently overcome at higher thermal energies. Similar temperature-dependent transport characteristics have been reported in semiconducting heterojunctions in which interfacial barriers or band offsets govern carrier dynamics.^40–42^

It should be emphasized, however, that nonlinear current–voltage characteristics in oxide heterostructures are not uniquely indicative of type-II band alignment. Alternative mechanisms, including contact-limited transport, barrier inhomogeneity, percolation effects associated with discontinuous morphologies, or Schottky-like behavior at the metal/oxide interface, may also contribute to rectifying *I*–*V* responses. In the absence of direct band alignment measurements, the type-II heterojunction model is therefore considered a plausible interpretation supported by transport trends rather than a directly demonstrated mechanism.

To further assess the nature of the interfacial transport, the activation energy (*E*_a_) associated with carrier conduction was extracted from temperature-dependent measurements by using Arrhenius analysis. This approach allows evaluation of effective interfacial barriers and changes in the dominant transport mechanism, such as transitions between thermally activated conduction and hopping processes.^43,44^ The corresponding Arrhenius plots are provided in the Supporting Information (Figures S1 and S2), and the extracted Ea values are summarized in Table 3.

**Table 3:** Activation Energy for Carrier Transport Through the Interface SnO_2_/TiO_2_, Obtained via Arrhenius Plot

sample’s Δ*H* (cm)	*Ea* (meV)
0.0	6.5 ± 2.3
0.3	3.65 ± 0.5
0.6	43.9 ± 15

Heterostructures with small activation energies (6,5 and 3.7 meV for Δ*H* = 0 and 0.3 cm) may show poorly defined interfaces that generate many shallow defects.^43–46^ These defects form localized states that enable thermally assisted tunneling or hopping, lowering the energy needed for charge transport.^43,44^ In contrast, the Δ*H* = 0.6 cm sample has a sharper interface, fewer shallow traps, and conduction dominated by electrons thermally released from deeper donor levels (≈50–60 meV, probably linked to Ti^3+^ in rutile TiO_2_). This observation results in a higher apparent activation energy. The coexistence of multiple trap states also explains the non-Arrhenius behavior, as different defects become active at different temperatures.^47^

For Δ*H* = 0 and 0.3 cm, deviations from exponential temperature dependence are observed below 200 K (Figure S2), indicating a weakly activated transport regime dominated by hopping or tunneling through shallow localized states.^43,44^ The near-linear increase of current with temperature in this range is characteristic of non-Arrhenius trap-assisted conduction. In contrast, the Δ*H* = 0.6 cm heterostructure exhibits an exponential temperature dependence of conductivity, consistent with thermally activated transport across a more defined interfacial barrier. The higher activation energy obtained for this sample suggests a reduced contribution from shallow defect states, which may arise from improved local interface definition or decreased barrier inhomogeneity. This behavior is compatible with models based on a staggered band alignment, even though it does not uniquely establish a specific band alignment mechanism.

During gelation, solvent volume reduction decreases the critical nucleus size and increases the particle deposition on the substrate. According to classical nucleation theory,^27^ stable nuclei form at a critical radius (r) corresponding to the maximum change in Gibbs free energy (Δ*G*). This reduction in r favors the formation and stabilization of smaller TiO_2_ clusters on the substrate surface.

As previously discussed, a reduced agglomerate size may promote densification of deposited films, yielding more compact and uniform morphologies. This structural uniformity is, likely, what improved the interface coherence between the TiO_2_ and SnO_2_ deposited layers, reducing interfacial defects and possibly lowering the energy barrier for charge transport. Smaller agglomerates also tend to increase surface contact and fixation during layer formation, enhancing film continuity.^31,48^ Moreover, localized interfacial states originating from residual agglomerates may act as hole-trapping centers, contributing to recombination pathways under an applied bias.^43–45^ These phenomena, collectively, help to explain the nonlinear and temperature-sensitive electrical characteristics observed in Figure 13 for the Δ*H* = 0.6 cm sample.

Solvent volume reduction during gelation leads to a decrease in nucleus size, while increasing the number of deposited particles on the substrate or previously deposited film. According to classical nucleation theory, particles tend to form stable structures at a critical radius (*r*) corresponding to the maximum variation in Gibbs free energy (Δ*G*), as described by Wu et al.^27^

6 $$\Delta G=-\frac{4{\pi r}^{3}}{3V}\Delta \mu +4{\pi r}^{2}\sigma$$

where *r* is the cluster radius, *V* the molar volume, Δμ the difference in chemical potential, and σ the surface tension across the spherical cluster.

As the solvent evaporates, the available volume for nucleation decreases, reducing the critical nucleus radius (*r*) as described by eq 6. The balance between bulk energy gain and surface energy cost determines nucleus stability, with smaller clusters exhibiting higher surface-to-volume ratios, which favors their attachment to existing solid surfaces rather than remaining suspended. Consequently, TiO_2_ nuclei preferentially fix onto the rutile SnO_2_ substrate, where structural compatibility and similar lattice parameters minimize interfacial strain and stabilize the deposited phase.

The preferential nucleation of rutile TiO_2_ arises from its structural compatibility with rutile SnO_2_, which minimizes strain energy and favors epitaxial-like growth.^12^ In contrast, anatase and brookite introduce a higher lattice mismatch and interfacial tension, making their formation less stable. As solvent evaporation constrains *r*, rutile-phase stabilization becomes more likely, yielding a more uniform TiO_2_/SnO_2_ interface.^12,16^ Smaller agglomerates further promote rutile formation by providing more nucleation sites and enabling growth at lower temperatures, enhancing charge-carrier separation and photocatalytic performance.^12,33^

In films grown with Δ*H* = 0.6 cm, smaller particle sizes promote denser packing during thermal annealing.^19^ This densification increases grain boundary scattering and resistivity, while reducing crystallite size, which lowers carrier concentration by limiting oxygen vacancies, the main source of n-type conductivity in TiO_2_.^34^ These structural changes also influence phase stability, as the crystallite size governs the formation of TiO_2_ polymorphs.^35–37^

## 4 Conclusions

The biphasic dip-coating method presented in this study introduces a new deposition parameter that can influence thin-film formation, affecting thickness control, homogeneity, surface roughness, topology, sintering behavior, and material packing. By modifying the height of the floating phase (Δ*H*), the method alters the draining regime, utilizing the surface tension of the upper phase to shift the stagnation point closer to the substrate’s surface. This shift promotes a capillary-driven drainage regime, typically associated with lower withdrawal speeds, thereby altering the surface morphology and particle packing. A more complete decoupling of Δ*H* from thickness effects will require thickness-matched samples prepared under different floating-phase conditions, which is currently beyond the scope of this study but represents an important direction for future work.

When applied to the heterostructure assembly, the method enhances interfacial compatibility by promoting rutile-phase nucleation during TiO_2_ deposition onto rutile SnO_2_, owing to their structural and dimensional similarities. As a result, the biphasic approach yields a more coherent and homogeneous interface, may influence phase evolution, consistent with reported size-dependent effects in sol–gel TiO_2_ systems and stabilization, and leads to transport behavior consistent with the formation of an effective heterojunction, compatible with type-II band alignment models reported in the literature.

Numerical simulations of the biphasic dip-coating process support these experimental findings, revealing that increasing Δ*H* narrows and lowers the liquid–liquid interface, altering the dominant region of fluid dynamics. At higher Δ*H*, the interface exhibits elevated pressure and velocity values, while at lower Δ*H*, these effects remain confined within the bulk liquid. The simulations highlight the critical roles of pressure-driven flow, surface tension, and substrate interaction in the governing film deposition. While modeling assumptions—such as neglecting evaporation, non-Newtonian effects, and nanoscale interactions—limit exact real-world applicability, the simulated data align well with the experimentally inferred flow regimes.

To further characterize the electrical behavior across the heterostructure interface, activation energies were extracted from Arrhenius plots. The more uniform interface observed at Δ*H* = 0.6 cm exhibited a higher activation energy, consistent with effective interfacial barriers with preserved band offsets and a lower density of shallow traps, leading to thermally activated transport via deeper donor levels. In contrast, the lower activation energies at Δ*H* = 0 and 0.3 cm reflect a higher density of interfacial disorder and shallow states that enable low-barrier conduction pathways.

These findings offer valuable insights into the design and fabrication of thin-film-based electronic devices, providing improved control over morphology, crystallinity, and interfacial properties. The versatility of the biphasic dip-coating method, particularly for engineering functional oxide heterostructures, emphasizes its potential for applications in optoelectronics, sensors, photovoltaics, catalysis, and transparent conductive coatings, warranting further exploration and process optimization in future studies.

## Supplementary Material



## Notes

Simple method for the analysis of the thermally activated behavior of the heterojunction, yielding the activation energy for interface carrier transport using an Arrhenius procedure, which provides insights into the dominant transport mechanism and Arrhenius plot and activation energy evaluation for samples with Δ*H* = 0, Δ*H* = 0.3, and Δ*H* = 0.6 cm, as well as figures of *I*/*I*_0_ as a function of temperature with exponential fit for the samples deposited with the same heights of the floating phase (PDF)
