# Supplementary material for: TiO2 Thin Films Obtained via Two-Phase Dip-Coating: Impact on Surface Roughness and Application to Heterostructures
Source: ACS Omega. 2026 Feb 10;11(7):11457–69. doi: 10.1021/acsomega.5c09163 (PMC12947213; doi:10.1021/acsomega.5c09163)
Supplement: Supplementary file 1 [file ao5c09163_si_001.pdf]

TiO_2 Thin Films obtained via Two-Phase Dip-Coating: Impact on Surface Roughness and Application to Heterostructures

Luiz Felipe Kaezmarek Pedrini, Natália Carli de Oliveira, Luis Vicente de Andrade Scalvi

Supplementary material

Evaluation of Activation Energy for electrical transport at the Interface

To evaluate the thermally activated behavior of the heterojunction, the activation energy *(Ea)* for carrier transport can be studied using an Arrhenius analysis [1,2]. This involves plotting the natural logarithm of the current (*ln I*) as a function of the inverse absolute temperature (*1/T*) at a fixed applied voltage. The temperature dependence of current in semiconducting junctions typically follows the Arrhenius equation:

$$I(T) = I0 \,.\, e^{-Ea/kT} \quad \text{(S1)}$$

Taking the natural logarithm of both sides yields a linear relationship:

$$\ln I = \ln I0 - \frac{Ea}{k}.\, 1/T \quad \text{(S2)}$$

From this expression, the slope of the linear region in the ln I vs. 1/T plot corresponds to –Ea/k, where k is Boltzmann's constant. By fitting a straight line to the data, the activation energy can be calculated as:

$$Ea = \; -k.slope \quad \text{(S3)}$$

This method provides insights into the dominant transport mechanism. A positive, thermally activated Ea indicates that charge carriers require a minimum thermal energy to overcome a potential barrier at the interface—consistent with behavior observed in type-II heterojunctions where interfacial band offsets play a critical role in controlling current flow.

The plot suggested by equation S2 as done in figure S1, allowing the evaluation of the activation energy (Ea). In this figure, the natural logarithm of the current (ln(I)) is

plotted as a function of the inverse temperature (1/T) for different values of ΔH (0, 0.3, and 0.6). The current values used correspond to current at 100V at each temperature. Linear fits (red lines) allow extraction of activation energy (Ea) from the slope. These results seem to point to a dependence of Ea on ΔH, with the system showing strong thermal activation behavior at the lowest ΔH value.

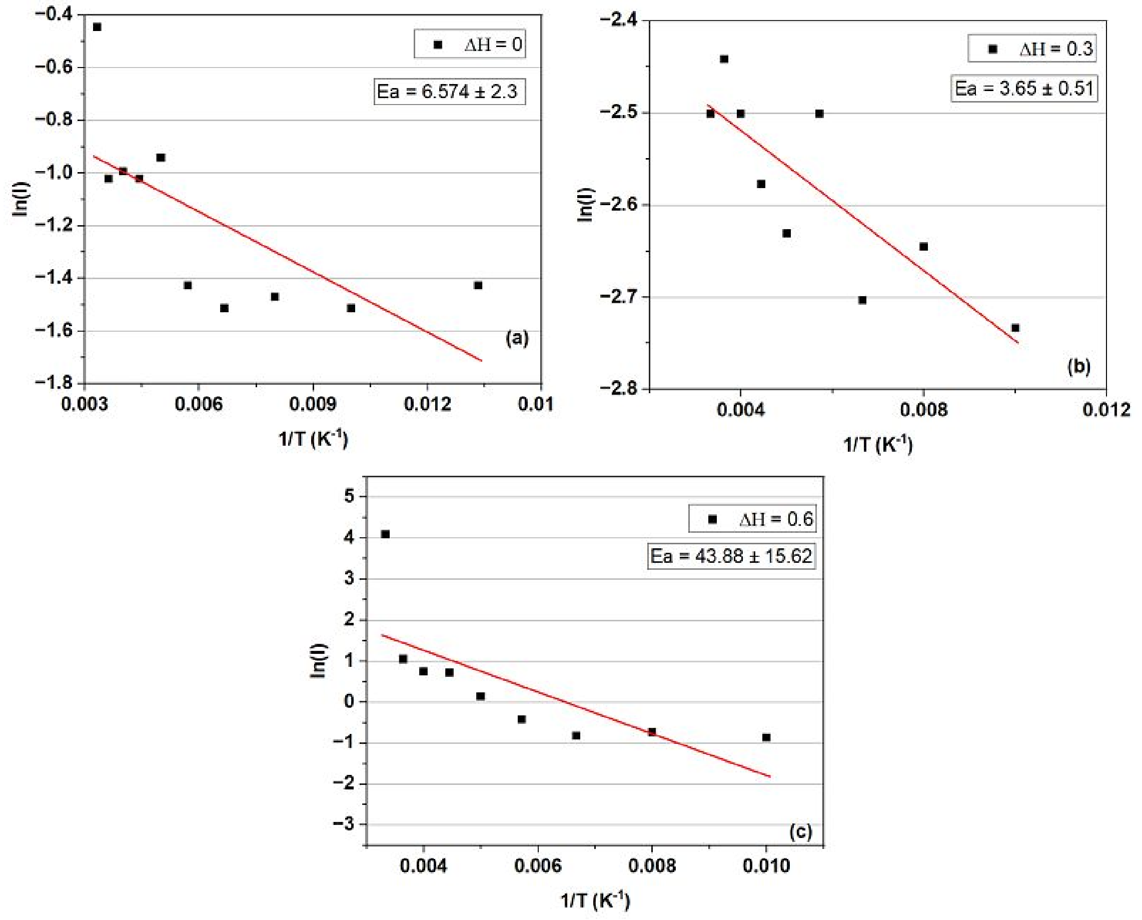

Figure S1: Arrhenius plot and activation energy evaluation for samples (a) ΔH = 0; (b) 0.3; (c) 0.6 cm

The Arrhenius model is widely used to describe thermally activated processes, in which the current depends exponentially on temperature. However, the application of this model assumes that charge transport is dominated by a single thermally activated mechanism, which is not always the case in complex systems, especially in thick films or in low-temperature regions (<200 K) [3]. In this context, a complementary analysis was proposed using an Arrhenius-like exponential fit as well as a linear model in two distinct ranges: below 200 K and thicker films (ΔH = 0 and ΔH = 0.3 cm), where the exponential fit presents systematic deviations and lower statistical quality and above 200 K.

To further investigate the temperature dependence of the current and evaluate deviations from the Arrhenius behavior, the normalized current (I/I_0) was plotted as a function of temperature (T) for all samples (Figure S2). The proposed fit relies on equations S4 and S5.

$$\frac{I(T)}{I0} = e^{-Ea/kT} \tag{S4}$$

$$\frac{I(T)}{I0} = \alpha T + \beta \tag{S5}$$

As can be seen in the images at the right side of figure S2, the fit based on a single exponential adjustment does not lead to a reliable fit, with a large dispersion of points. On the other hand, the fit based on two linear regions highlights where the current increases linearly with temperature, suggesting a weaker thermal activation process. Such behavior becomes particularly evident for the films deposited with $\Delta H = 0$ and 0.3 cm and at low temperatures (< 200 K), where the exponential Arrhenius fit shows systematic deviations.

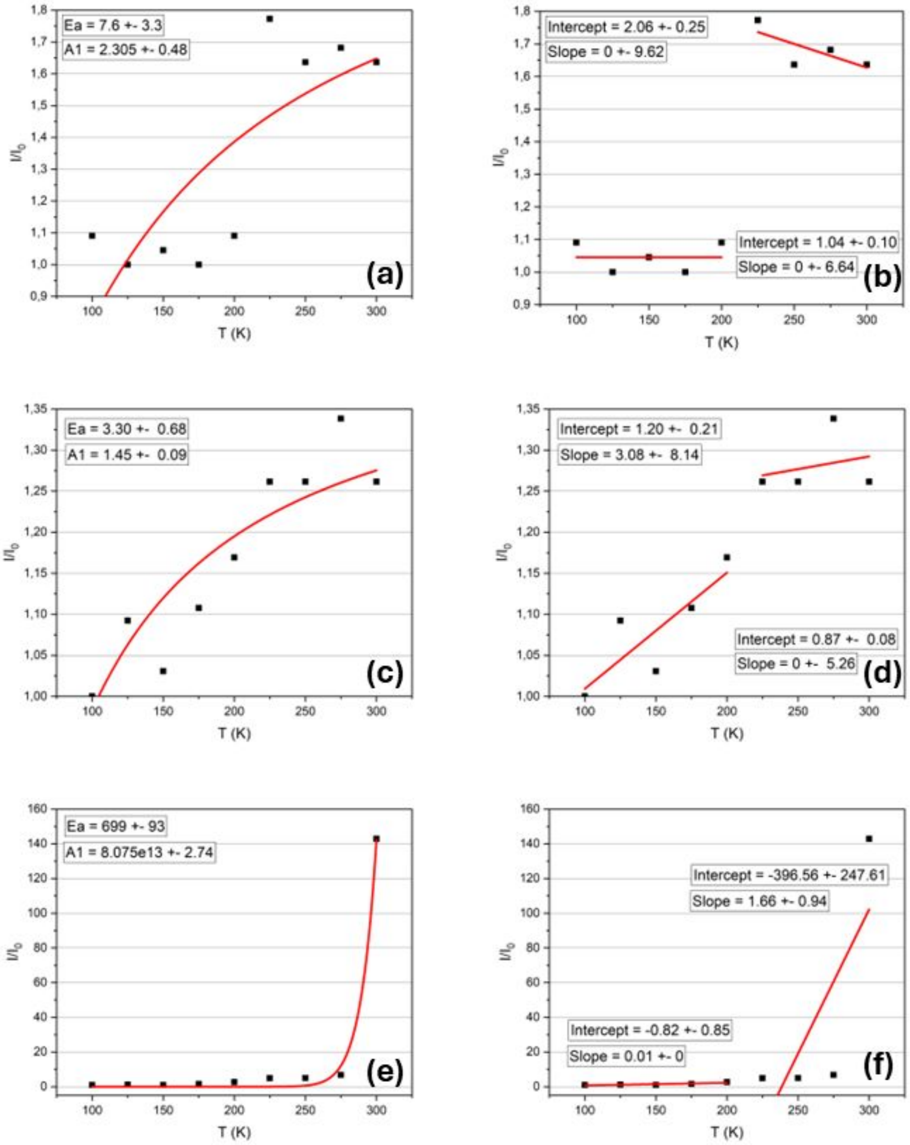

Figure S2: I/I_0 x Temperature with exponential fit according to Arrhenius plot for samples (a) ΔH = 0; (c) 0.3 cm; (e) 0.6 cm; and by linear plot for two regions to samples (b) ΔH = 0; (d) 0.3 cm ;(f) 0.6 cm.

The change in mathematical regime in the conduction of films may indicate that other mechanisms control transport, such as for thicker films (ΔH = 0 and 0.3), where transport may be mainly dominated by tunneling or hopping between shallow traps, resulting in lower effective energy for carriers to overcome the interface barrier [4,5]. Structural changes associated with film thickness may be also accounted for, which is regulated by the floating phase, as in the case of samples with ΔH = 0.6 cm for instance, since the current increases with temperature in a manner consistent with a junction-mediated transport mechanism for this sample, suggesting that there is a potential barrier

at the interface and that this is efficiently overcome with increasing temperature in the system.

Thus, the linear behavior observed in low temperature ranges may indicate a break in the Arrhenius dependence, suggesting that transport is dominated by mechanisms that are not purely thermally activated. The most likely scenario is hopping between shallow traps in which carriers move between energetically favorable localized states without the need to overcome the entire potential barrier [6]. Under this condition, electrical conductivity shows a weak dependence on temperature, resulting in a more gradual increase in transport as the temperature rises [3,5].

Although linear fitting provided a reasonable description of the temperature-dependent behavior, particularly highlighting a change in slope near 200 K, the exponential model yielded a better representation for the $\Delta H = 0.6$ cm sample (fig. S2e). In this case, the conductivity increased more sharply with temperature, consistent with an exponential dependence rather than a segmented linear trend, suggesting a transition to a regime dominated by thermally activated processes.

(1) Schipani, F.; Aldao, C. M.; Ponce, M. A. Schottky Barriers Measurements through Arrhenius Plots in Gas Sensors Based on Semiconductor Films. *AIP Adv.* **2012**, *2* (3), 032138. https://doi.org/10.1063/1.4746417.

(2) Haidry, A. A.; Durina, P.; Tomasek, M.; Gregus, J.; Schlosser, P.; Mikula, M.; Truhly, M.; Roch, T.; Plecenik, T.; Pidik, A.; Zahoran, M.; Kus, P.; Plecenik, A. Effect of Post-Deposition Annealing Treatment on the Structural, Optical and Gas Sensing Properties of TiO_2 Thin Films. *Key Eng. Mater.* **2012**, *510–511*, 467–474. https://doi.org/10.4028/www.scientific.net/KEM.510-511.467.

(3) Yildiz, A.; Lisesivdin, S. B.; Kasap, M.; Mardare, D. Electrical Properties of TiO2 Thin Films. *J. Non-Cryst. Solids* **2008**, *354* (45–46), 4944–4947. https://doi.org/10.1016/j.jnoncrysol.2008.07.009.

(4) Mora-Fonz, D.; Kaviani, M.; Shluger, A. L. Disorder-Induced Electron and Hole Trapping in Amorphous TiO 2. *Phys. Rev. B* **2020**, *102* (5), 054205. https://doi.org/10.1103/PhysRevB.102.054205.

(5) Bharadwaja, S. S. N.; Venkatasubramanian, C.; Fieldhouse, N.; Ashok, S.; Horn, M. W.; Jackson, T. N. Low Temperature Charge Carrier Hopping Transport Mechanism in Vanadium Oxide Thin Films Grown Using Pulsed Dc Sputtering. *Appl. Phys. Lett.* **2009**, *94* (22), 222110. https://doi.org/10.1063/1.3139864.

(6) Hill, R. M. Variable-Range Hopping. *Phys. Status Solidi A* **1976**, *34* (2), 601–613. https://doi.org/10.1002/pssa.2210340223.
